# Neuromodulator regulation and emotions: insights from the crosstalk of cell signaling

**DOI:** 10.3389/fnmol.2024.1376762

**Published:** 2024-03-07

**Authors:** Daisuke Tsuboi, Taku Nagai, Junichiro Yoshimoto, Kozo Kaibuchi

**Affiliations:** ^1^Division of Cell Biology, International Center for Brain Science, Fujita Health University, Toyoake, Aichi, Japan; ^2^Division of Behavioral Neuropharmacology, International Center for Brain Science, Fujita Health University, Toyoake, Aichi, Japan; ^3^Department of Biomedical Data Science, Fujita Health University School of Medicine, Toyoake, Aichi, Japan

**Keywords:** neuromodulator, cell signaling, emotional behavior, KiOSS, protein database

## Abstract

The unraveling of the regulatory mechanisms that govern neuronal excitability is a major challenge for neuroscientists worldwide. Neurotransmitters play a critical role in maintaining the balance between excitatory and inhibitory activity in the brain. The balance controls cognitive functions and emotional responses. Glutamate and γ-aminobutyric acid (GABA) are the primary excitatory and inhibitory neurotransmitters of the brain, respectively. Disruptions in the balance between excitatory and inhibitory transmission are implicated in several psychiatric disorders, including anxiety disorders, depression, and schizophrenia. Neuromodulators such as dopamine and acetylcholine control cognition and emotion by regulating the excitatory/inhibitory balance initiated by glutamate and GABA. Dopamine is closely associated with reward-related behaviors, while acetylcholine plays a role in aversive and attentional behaviors. Although the physiological roles of neuromodulators have been extensively studied neuroanatomically and electrophysiologically, few researchers have explored the interplay between neuronal excitability and cell signaling and the resulting impact on emotion regulation. This review provides an in-depth understanding of “cell signaling crosstalk” in the context of neuronal excitability and emotion regulation. It also anticipates that the next generation of neurochemical analyses, facilitated by integrated phosphorylation studies, will shed more light on this topic.

## 1 Introduction

**Figure 1:**
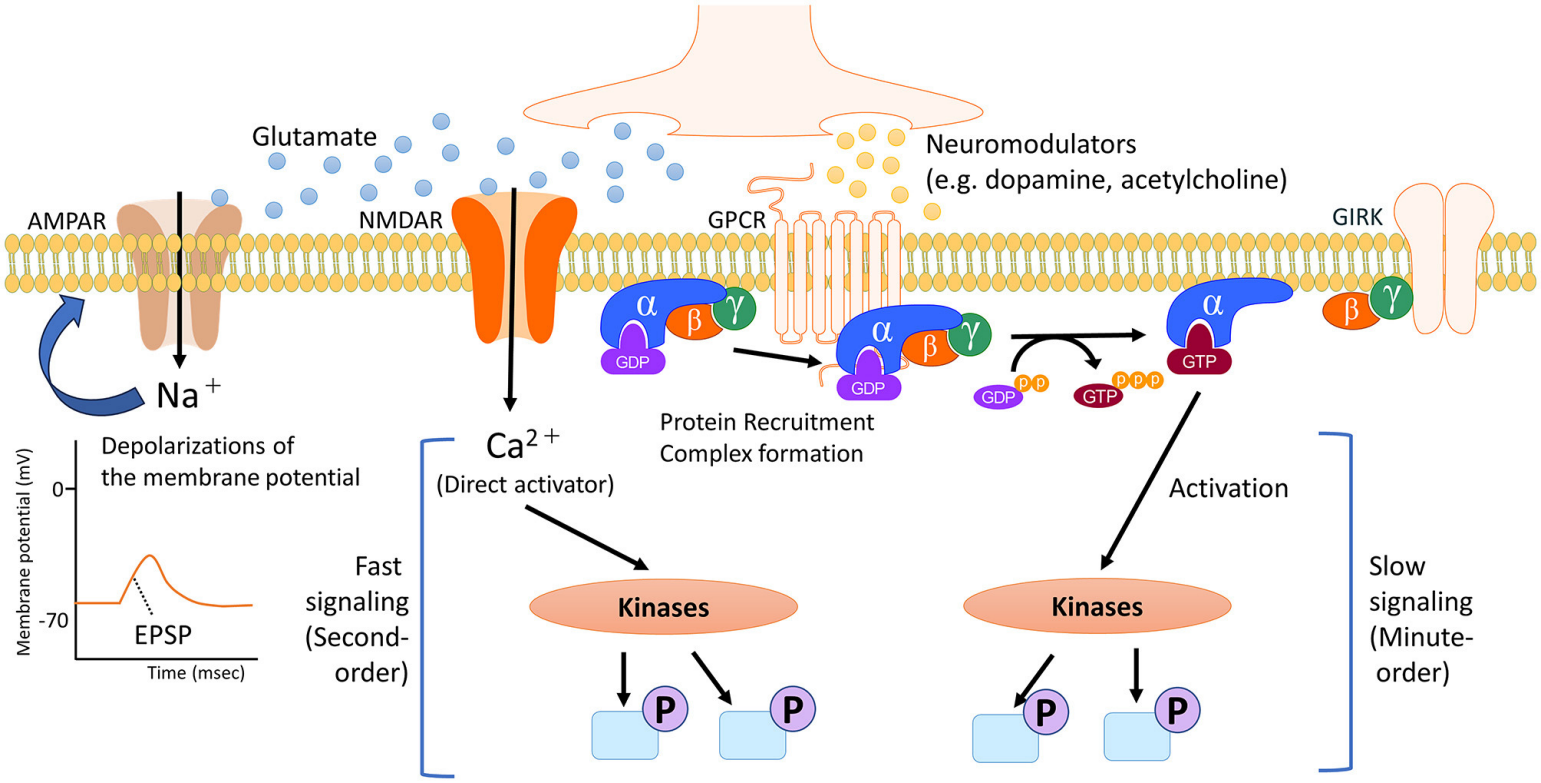
Timescale of cell signaling regulated by neurotransmitters and neuromodulators. In the brain, neurotransmitters and neuromodulators work as triggers activating several cell signaling pathways, depending on the specific receptor. Neurotransmitters like glutamate primarily initiate the activity of signaling pathways through direct ionic interactions. In addition, the flow of positively charged ions into post-synapses causes temporary depolarization of the postsynaptic membrane potential, namely excitatory postsynaptic potential (EPSP). Conversely, neuromodulators such as dopamine and acetylcholine undergo multiple processes, including the protein recruitment and complex formation of the G protein. G proteins exist as heterotrimers consisting of α, β and γ subunits. The α subunit is bound to GDP. When an agonist binds to a GPCR, which catalyzes the exchange of GDP for GTP on the α subunit. This causes a conformational change in the α subunit, leading to its dissociation from the βγ dimer. The dissociated subunits act as signal transducers. These complex steps for activating intracellular signaling contribute to slower and longer-lasting effects that spread out more broadly. NMDAR, N-methyl-D-aspartate (NMDA)-type glutamate receptor; AMPAR, α-amino-3-hydroxy-5-methyl-4-isoxazolepropionic acid (AMPA)-type glutamate receptor; GPCR, G protein-coupled receptor; GIRK, G protein-gated inwardly rectifying potassium channel; GDP, guanosine diphosphate; GTP, guanosine triphosphate.

Regulators of neural circuits are primarily chemicals that act as neurotransmitters and neuromodulators. The roles of these receptors are determined by the receptors to which they bind and their impact on synaptic transmission and cell signaling. Receptors for neurotransmitters and neuromodulators can be either ligand-activated ion channels (called ionotropic receptors) or G-protein-coupled receptors (also called metabotropic receptors). While ionotropic receptors respond rapidly to ligands by opening or closing ion gates, metabotropic receptors produce slower effects by influencing trimeric G-proteins, enzymes, and channels in the membrane. Neurotransmitters, though not all, contribute to the regulation of neuronal excitability through rapid ionotropic effects. Conversely, neuromodulators have slower, longer-lasting, and more diffuse effects on neurophysiology, often due to their actions at metabotropic receptors (Figure 1, Baskys, 1992; Burrows and Oxford Scholarship Online, 1996). The activation of neuromodulator receptors does not directly trigger neuronal excitation or inhibition, but it modulates transmission to and through a region by physiologically altering cellular properties such as release probability and pyramidal cell adaptation, thus influencing overall information transmission (Nadim and Bucher, 2014).

The concept of excitatory/inhibitory (E/I) balance refers to maintaining a healthy balance between the signals that activate or inhibit neurons. In a healthy brain, there is a delicate balance between these two types of signals (Froemke, 2015). Excitatory signals, typically carried by the neurotransmitter glutamate, stimulate neurons and promote neuronal activity. Conversely, inhibitory signals, typically mediated by the neurotransmitter γ-aminobutyric acid (GABA), dampen neuronal activity and help regulate the overall level of excitation. Glutamate is the primary excitatory neurotransmitter in the adult mammalian brain and plays pivotal roles in numerous processes. Its release into the synaptic cleft is driven by neuronal depolarization and is calcium-dependent (Zhou and Danbolt, 2014). α-amino-3-hydroxy-5-methyl-4-isoxazolepropionic acid (AMPA)-type glutamate receptors (AMPARs) are essential for rapid excitatory neurotransmission and are crucial for mediating excitatory postsynaptic currents (EPSCs) and potentials (EPSPs). N-methyl-D-aspartate (NMDA)-type glutamate receptors (NMDARs) are blocked by magnesium ions (Mg^2+^). Upon depolarization by AMPAR-mediated EPSPs, the Mg^2+^ block is removed, allowing NMDARs to become the primary pathway for calcium ion (Ca^2+^) influx. This Ca^2+^ influx through NMDARs triggers responses like synaptic plasticity in the cells. When glutamate is released into the synaptic cleft, it activates AMPARs, leading to the rapid rise and decay of EPSCs and EPSPs (Zhou and Hablitz, 1998). This process directly contributes to neuronal excitability, promoting the generation of action potentials. GABA is the major inhibitory neurotransmitter in the adult brain. It plays a critical role in regulating neuronal excitability and maintaining the E/I balance in the brain. GABA acts primarily through two types of receptors: ionotropic receptors (type A, GABA-A; non-canonical type A, GABA-A-rho) and metabotropic type B (GABA-B) receptors (Hill and Bowery, 1981; Bormann, 1988; Mohler, 1989; Zhang et al., 2001). GABA-A receptors are ligand-gated ion channels composed of multiple subunits. When GABA binds to the GABA-A receptor, chloride ion channels open, resulting in an influx of chloride ions into the neuron. This influx of negatively charged chloride ions hyperpolarizes the neuron, decreasing the likelihood of generating an action potential. This hyperpolarization inhibits neuronal firing and reduces neuronal excitability. As G-protein-coupled receptors, GABA-B receptors provide important mechanisms for regulating neuronal excitability through the modulation of ion channels, neurotransmitter release, and membrane potentials. Thus, activation or inhibition of GABA-B receptors contributes to the fine-tuning of neuronal activity in the brain.

Disruption of the E/I balance can lead to problems in neural signaling and contribute to the development of various neurodevelopmental and neuropsychiatric disorders. Such imbalances may impact the connectivity and function of specific brain regions involved in cognitive processes, emotional regulation, and mood control. Indeed, a dysregulated E/I balance is a significant characteristic of autism spectrum disorder (ASD) and schizophrenia (Tatti et al., 2017). In ASD, there is evidence of increased excitatory neurotransmission and decreased inhibitory neurotransmission, resulting in an overall increase in the ratio of excitation to inhibition (Rubenstein and Merzenich, 2003; Sohal and Rubenstein, 2019; Hollestein et al., 2023). Schizophrenia is also associated with dysregulation of E/I balance (Foss-Feig et al., 2017). Such pathophysiology of ASD and schizophrenia may involve impaired neuromodulators, but the molecular basis of neuromodulation remains poorly understood. This review discusses how neuromodulators control neuronal excitation from the perspective of intracellular signaling. In addition, the last section of the review mentions that the next generation of neurochemical analyses, facilitated by integrated phosphorylation studies, will shed more light on the molecular machinery underlying the regulation of neuronal excitability by neuromodulators.

## 2 Molecular mechanisms of neuronal excitability by dopamine and its contribution to cognitive and emotional regulation

Neuromodulators, such as dopamine, acetylcholine, and serotonin, are crucial in modulating the excitability of certain neurons. This modulation affects cognitive processes such as emotion and certain types of memory. Dopamine, a monoamine neuromodulator, is involved in motor function, motivation, working memory, reward-related learning, and orofacial movements (Girault and Greengard, 2004; Li et al., 2023). Dysregulated dopamine signaling has been implicated in various neuropsychological disorders, including Parkinson's disease, drug addiction, impulsive behavior, attention-deficit/hyperactivity disorder, ASD, and schizophrenia (Carlsson, 2001; Hyman et al., 2006; Iversen and Iversen, 2007; Swanson et al., 2007; Koob and Volkow, 2010). Dopamine neurons are mainly located in two areas of the mammalian brain: the substantia nigra (SN) and the ventral tegmental area (VTA) (Luo and Huang, 2016). These dopaminergic neurons form the distinct pathways play critical roles in several brain functions. The nigrostriatal pathway, which originates from SN neurons, primarily projects to the dorsal striatum and controls postural reflexes, and motor initiation (Prensa and Parent, 2001; Jin and Costa, 2010). VTA neurons give rise to two pathways, the mesolimbic and mesocortical pathways (Islam et al., 2021). The mesolimbic pathway projects to the subcortical and limbic nuclei, including the nucleus accumbens (NAc). This pathway plays a significant role in emotional behaviors like reward, aversive behaviors and drug abuse (Nestler and Carlezon, 2006; De Jong et al., 2019). The mesocortical pathway projects to the cingulate cortex, entorhinal cortex, and medial prefrontal cortex and is involved in cognitive processes and goal-directed behaviors (Carr and Sesack, 2000; Naneix et al., 2012; Verharen et al., 2018). Therefore, dopamine regulates different physiological functions depending on the region of the brain to which it projects.

The dorsal striatum and NAc are subcortical regions of the brain that play crucial roles in various behavioral processes, including motor control, motivation, reward, and learning. These two regions are abundantly innervated by glutamatergic projections from the cerebral cortex. Dopamine modulates the glutamatergic corticostriatal pathway by altering the excitability of dorsal striatal neurons. Dopamine receptors are classified into two groups: D1-class receptors (D1 and D5) and D2-class receptors (D2, D3, and D4). The specific effects of dopamine on excitatory and inhibitory signals depend on the cell types expressing these receptors (Missale et al., 1998). D1-like receptors, when activated by dopamine, activate stimulatory Gs/olf proteins. This activation leads to an increase in intracellular cyclic adenosine monophosphate (cAMP) levels, which can enhance excitatory signals in the neural circuit (Nishi et al., 2000; Scheggi et al., 2018). However, D2-like receptors activate inhibitory Gi/o proteins. This activation leads to a decrease in cAMP levels, which can reduce excitatory signals and promote inhibitory signals in the neural circuit (Rocheville et al., 2000; Bozzi and Borrelli, 2013; Polit et al., 2020). This fluctuating level of cAMP regulates the activity of signal transduction pathways, including Protein kinase A (PKA). Consequently, dopamine can influence the balance between excitatory and inhibitory transmission in the dorsal striatum and other brain regions by modulating the activity of the PKA pathway (Gerfen and Surmeier, 2011; Zhang et al., 2022). The neurons comprising the dorsal striatum are primarily GABAergic medium spiny neurons (MSNs) that express either dopamine D1 receptors (D1R-MSNs) or dopamine D2 receptors (D2R-MSNs). D1R-MSNs and D2R-MSNs play distinct roles in behavior (Hikida et al., 2010). Maintaining a balance between D1R-MSN and D2R-MSN activity in the dorsal striatum is crucial for the cooperative execution of two distinct pathways: the direct pathway and the indirect pathway (Mink and Thach, 1993; Macpherson et al., 2014; Fujita and Eidelberg, 2017; Lee et al., 2021). Stimulation of the direct pathway (the pathway consisting of D1R-MSNs) activates motor behaviors (Kravitz et al., 2010; Tecuapetla et al., 2016; Lee et al., 2021). The direct pathway initiates with excitatory projections from the cortex to the dorsal striatum, which then sends inhibitory projections to the internal globus pallidus (GPi) (Gerfen and Surmeier, 2011). The GPi normally inhibits the thalamus, so inhibiting the GPi disinhibits the thalamus, which ultimately facilitates movement by exciting the motor cortex. On the other hand, stimulation of the indirect pathway (the pathway consisting of D2R-MSNs) inhibits motor activity (Sano et al., 2013). The indirect pathway also starts with excitatory projections from the cortex to the dorsal striatum, but then projects to the external globus pallidus (GPe) (Gerfen and Surmeier, 2011). The GPe inhibits the subthalamic nucleus, which excites the GPi. Thus, the net effect of the indirect pathway is to increase the inhibition of the thalamus, thereby suppressing competing motor programs to allow the selected movement to proceed (Kravitz et al., 2010; Gerfen and Surmeier, 2011). The balance between facilitating desired movements via the direct pathway and suppressing undesired movements via the indirect pathway allows for refined control of voluntary movement (Acharya and Kim, 2021). Silencing each pathway has the opposite effect. This balance ensures the fine-tuning of motor responses and prevents excessive or inappropriate movements. Cell signaling is a cellular process that involves a dynamic cycle of phosphorylation and dephosphorylation (Salazar and Hofer, 2006). The phosphorylation cycle controls cellular responses and maintains homeostasis. In the dorsal striatum, Paul Greengard and his colleagues demonstrated that PKA and dopamine- and cAMP-regulated phosphoprotein of 32 kDa (DARPP-32) in dorsal striatum are key players in the signaling pathways that regulate voluntary movement and emotional behaviors (Walaas et al., 1983; Scheggi et al., 2018). When dopamine release is enhanced, PKA is activated via D1Rs, leading to the phosphorylation of DARPP-32 at threonine 34 (Thr34) residue for the inhibition of protein phosphatase 1 (PP1). The inhibition of PP1 contributes to the stable phosphorylation of many PKA-substrates. However, proteins directly phosphorylated by PKA were not identified until the development of a comprehensive phosphoproteomic analysis.

## 3 Phosphoproteomics for evaluating the physiological significance of dopamine signaling

The development of proteomics analysis over the past two decades has been underpinned by large-scale protein studies using mass spectrometry. The integration of liquid chromatography and mass spectrometry (LC–MS/MS) analysis allows for the identification of numerous peptides, aiding protein identification (Link et al., 1999; Washburn et al., 2001). Proteome-wide studies of phosphoproteins have garnered increased attention because protein kinases play pivotal roles in various biological reactions and pathological conditions. Innovations in the enrichment of protein phosphorylation have contributed significantly to the elucidation of phosphorylation-dependent pathways (Oda et al., 2001; Blacken et al., 2006), and isotope labeling methods for quantitative analysis (Ong et al., 2002; Thompson et al., 2003; Ross et al., 2004) and many other technologies and methodologies have been introduced. Consequently, more than 200,000 phosphorylation sites have been characterized (Savage and Zhang, 2020). These results cannot be resolved by individual studies and require large-scale *in silico* data analysis. Therefore, future data-driven studies will explore the relationships among phosphorylation patterns and signaling molecules. However, much time and effort are expected to be required to establish causal relationships between protein kinases and their substrates.

**Figure 2:**
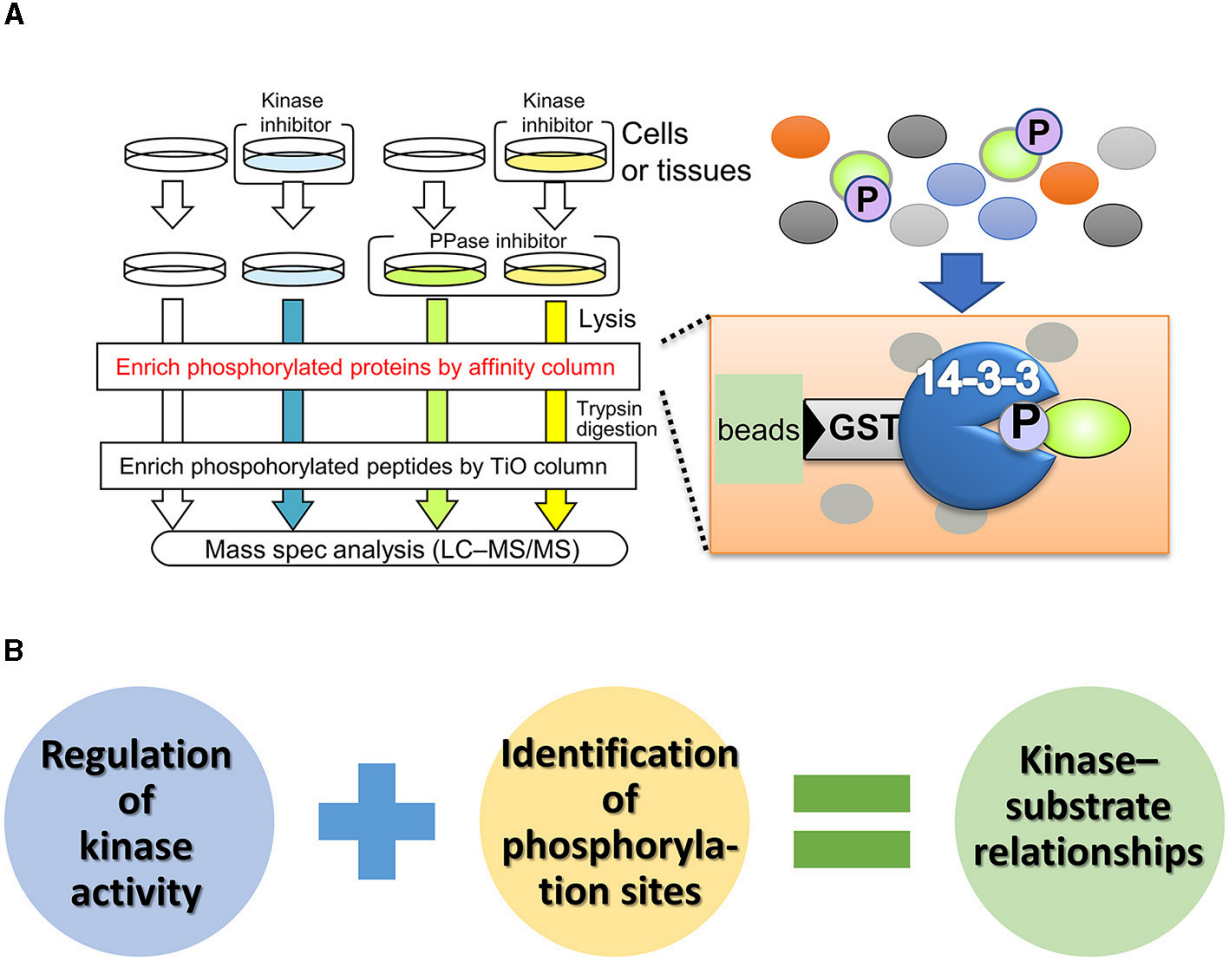
Outline and benefit of kinase-oriented substrate screening (KiOSS). **(A)** Schematic diagram of KiOSS for the identification of phosphoproteins and their phosphorylation sites. Protein phosphatase (PPase) inhibitors and a kinase inhibitor are applied to cells/tissues in culture, followed by protein extraction. Subsequently, phosphoproteins are concentrated using an affinity column that interacts with phosphoprotein-related proteins/domains, such as 14-3-3. This step is followed by trypsin digestion and subsequent enrichment of the phosphopeptides. Finally, protein phosphorylation is detected and measured via liquid chromatography tandem mass spectrometry (LC–MS/MS). **(B)** Combination of proteomics and pharmacological modulation. The combined proteomic approach allows us to identify the phospho-candidates and their phosphorylation sites for any kinase.

In contrast to unbiased proteomics, focused proteomic studies, which target specific molecules or protein subpopulations, have been widely reported (Kirkpatrick et al., 2013). Affinity purification of phosphoproteins using phosphorylation motif antibodies is a technique for identifying phosphorylation substrates of protein kinases (Gafken and Lampe, 2006; Edbauer et al., 2009; Wang et al., 2016). One of the substrate specificities of protein kinases is highly dependent on the amino acid sequence around the phosphorylation site (also known as the consensus sequence). Phosphorylation motif antibodies recognize and bind to phosphorylated serine/threonine residues and their surrounding sequences. The immunoprecipitates from these antibodies contain numerous phosphoproteins, which can be identified by mass spectrometry, allowing a comprehensive search for phosphorylated substrates. Although this method is simple, some kinases have low specificity for the phosphorylation consensus sequence, making it difficult to distinguish between kinases with similar phosphorylation sites and determine the upstream kinase responsible for substrate modification. To enrich phospho-substrates for specific kinases *in vivo*, Nishioka et al. (2012, 2019) developed a phosphorylated substrate screening method called kinase-oriented substrate screening (KiOSS) (Figure 2). KiOSS utilizes two technical features. The first is the use of phosphatase inhibitors in combination with kinase inhibitors. Phosphatase inhibitors can increase the basal phosphorylation level of substrates, including substrates phosphorylated, only under certain conditions, and require “priming phosphorylation” by other kinases. Under conditions of increased phosphorylation, pretreatment with kinase-specific inhibitors and subsequent LC–MS/MS phosphoprotein screening will allow high-performance detection. The second technical feature is the use of affinity columns coated with 14-3-3 proteins, FHA (forkhead associated), or WW (tryptophan-tryptophan) phosphorylation binding motifs to enrich phosphorylated peptides (Shohag et al., 2015). These proteins and motifs recognize and bind phosphorylated serine/threonine residues. The phosphorylation sites recognized by these motifs are involved in and regulate various cellular processes, including cell division, signal transduction, and cell polarization (Obsil and Obsilova, 2011). Hence, these phosphorylation-supplemented proteins/motifs are expected to enrich components of signaling cascades and dynamic phosphoproteins, rather than housekeeping and/or structural proteins.

**Figure 3:**
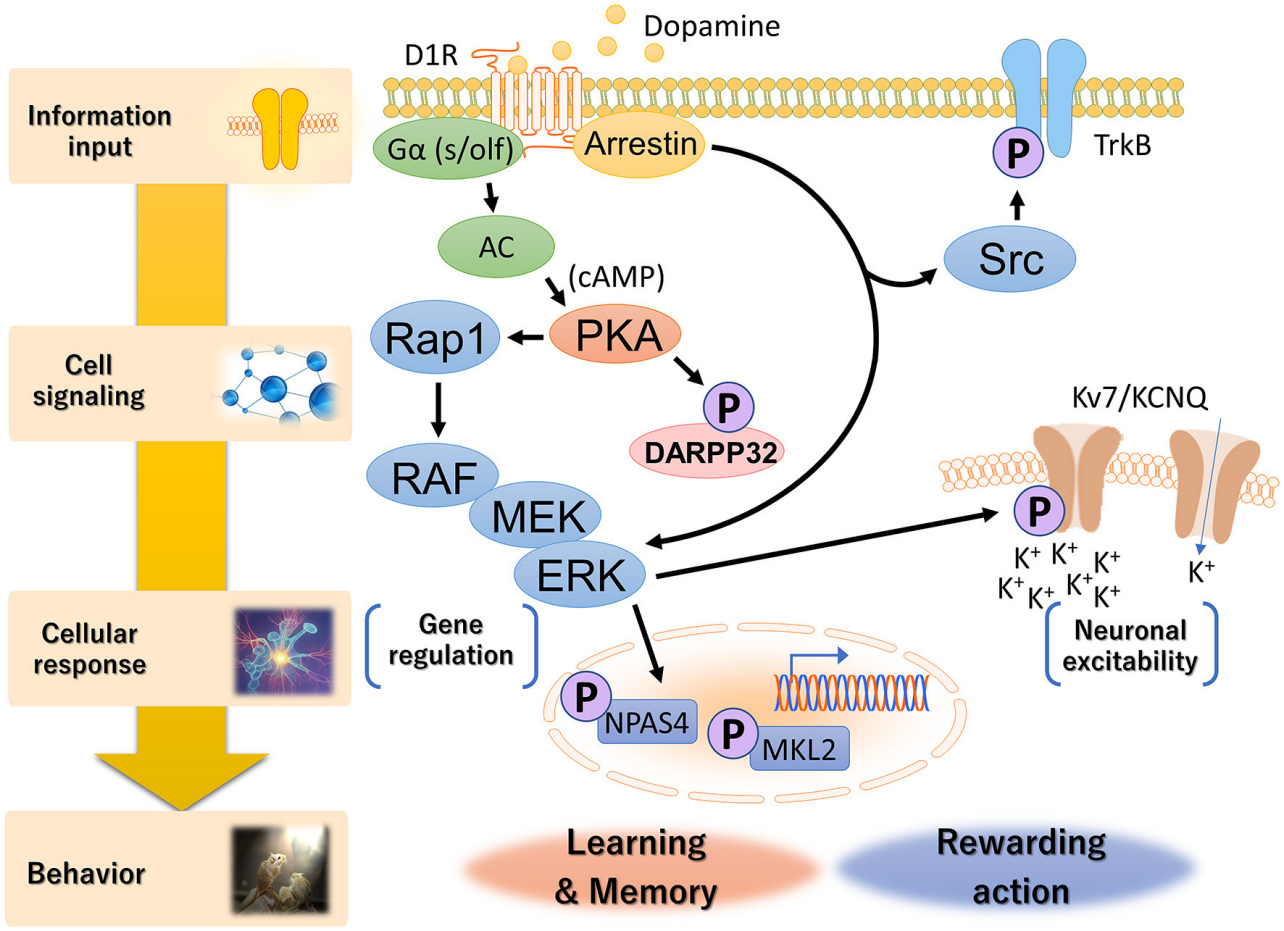
Hierarchical diagram of D1R signaling in the dorsal striatum and NAc. Dopamine is involved in a wide range of biological processes related to reward behavior, including reward prediction, learning, memory and addiction. D1Rs transduce downstream signals in G-protein-dependent and G-protein-independent manners. Canonical G-protein-based signaling activates PKA via increased cAMP production by adenylyl cyclase, followed by the Rap1–MAPK cascade. Non-canonical D1R–arrestin signaling increases the phosphorylation of Src and ERK, although the molecular mechanism is not fully understood. Activated ERK conducts distinct cellular responses, such as neuronal excitability and gene regulation, via specific phospho-substrates.

The KiOSS method has proven particularly useful for understanding the function of dopamine and identifying the target substrates of PKA. For instance, using this method, more than 100 new PKA substrates downstream of dopamine, including Rasgrp2 and Rap1gap, have been identified (Nagai et al., 2016a,b). Rasgrp2 and Rap1gap function as regulators of the small G protein Rap1. Guanine nucleotide exchange factors (GEFs) and GTPase-activating proteins (GAPs) are key regulators of small G-proteins, with GEFs catalyzing the activation of G-proteins by replacing bound GDP with GTP and GAPs enhancing GTPase activity to turn off G-proteins. PKA phosphorylates Rasgrp2 to increase GEF activity to Rap1, while the phosphorylation of the Rap1gap decreases GAP activity to Rap1. This dual pathway efficiently activates Rap1. Rap1 activates B-RAF (MAP3K1, MAPKKK), which in turn stimulates the MAPK signaling cascade. Nagai et al. (2016a) investigated the role of the Rap1–MAPK pathway in reward behavior using adeno-associated virus gene transfer. Expression of constitutively active forms of Rap1 and MEK1 (MAP2K1, MAPKK) in accumbal D1R-MSNs increased the excitability of D1R-MSNs and enhanced cocaine-induced place preference. In contrast, the opposite effect was induced by the expression of the dominant-negative MEK1 or knockout of the *Rap1* gene in D1R-MSNs. When the dominant-negative MEK1 was simultaneously expressed with constitutively active PKA or Rap1 in D1R-MSNs, the dominant-negative MEK1 attenuated the increase in susceptibility to cocaine reward induced by the constitutively active PKA- or Rap1. In the context of DARPP-32, the phosphorylation of Rasgrp2 induced by D1R activation is under the control of the DARPP-32–PP1 pathway (Kuroiwa et al., 2023). This interaction between the DARPP-32–PP1 and Rasgrp2–Rap1 signaling pathways is crucial for efficient D1R–Rap1 signaling in the dorsal striatum and is required for the dopamine action that regulates the neuronal functions of D1R-MSNs and the pathophysiology of neuropsychiatric disorders. These observations suggest the presence of a Rap1–MAPK signaling pathway downstream of D1R signaling and indicate that activation of this pathway enhances the excitability of D1R MSNs in the NAc (Figure 3).

## 4 Molecular mechanisms involved in reward-based learning behaviors

What is the function of MAPK/ERK in neuronal excitability? The D1R phosphoproteome has identified a voltage-gated potassium channel subunit, KCNQ2, as a phosphorylation substrate of ERK2 (Figure 3, Tsuboi et al., 2022). KCNQ potassium channels, also known as M-channels and Kv7.2/7.3/7.5, play a critical role in stabilizing neuronal excitability, thus influencing the E/I balance in the brain (Soh et al., 2014; Niday et al., 2017; Stincic et al., 2021). M-channels work by allowing potassium ions to flow out of neurons, which helps to suppress excessive neuronal firing and maintain a stable resting membrane potential (Brown, 1988; Cooper and Jan, 2003). The E/I balance is crucial for normal brain function, and disruption of this balance can lead to neurological disorders, including epilepsy. In the context of epilepsy, mutations in the genes encoding KCNQ channels have been associated with benign familial neonatal seizures and other forms of epilepsy. These mutations are believed to disrupt the normal function of the channels, affecting the E/I balance and causing seizures. Therefore, KCNQ channels contribute to this balance by inhibiting excessive neuronal activity. Tsuboi et al. (2022) demonstrated that activation of D1R inhibits KCNQ-mediated currents and increases D1R-MSN firing rates in NAc slices, which is abolished by ERK inhibition. KCNQ2 is phosphorylated downstream of dopamine signaling in NAc slices. Conditional deletion of *Kcnq2* in D1R-MSNs reduces the inhibitory effect of D1R activation on KCNQ channel activity while enhancing neuronal excitability and cocaine-induced place preference. These effects are restored by expression of wild-type, but not phospho-deficient KCNQ2. Hence, these phosphorylation events facilitate the neuronal excitability of D1R-MSNs in the NAc and regulate reward-related behavior (Tsuboi et al., 2022). The ERK–KCNQ2 signaling pathway plays roles in neuronal excitability and reward-related behavior. Choi et al. (2018) revealed that schizophrenia-related chromosomal deletion leads to abnormal dopaminergic modulation of prefrontal cortical interneuron activity, which is dependent on KCNQ2. Choi's group proposed that this abnormal modulation may contribute to the E/I imbalance observed in schizophrenic patients. These findings suggest that targeting KCNQ2 could be a potential therapeutic intervention for psychiatric disorders.

Learning accompanied by reward signals is considered one form of reinforcement learning and is known to aid in long-term memory retention, suggesting that the dopamine signaling pathway regulates gene expression. A downstream kinase for dopamine signaling, such as MAPK/ERK, regulates gene expression through nuclear translocation and phosphorylation of transcription factors (Treisman, 1996). Although the gene expression of brain-derived neurotrophic factor (BDNF) and cFOS in the dorsal striatum is induced by administration of cocaine (Larson et al., 2010), how reward signals regulate the expression of cellular activity factors, such as BDNF and cFOS, has not been determined. Recently, Funahashi et al. (2019) and Ariza et al. (2021) performed a proteomic analysis using affinity beads coated with cAMP response element-binding protein (CREB)-binding protein (CBP), a transcriptional coactivator involved in reward-related behavior. CBP-affinity chromatography and mass spectrometry identified more than 40 CBP-interacting proteins, including neuronal PAS domain-containing protein 4 (NPAS4) and megakaryoblastic leukemia 2 (MKL2) (Figure 3). NPAS4 is induced specifically in excitatory neurons upon calcium influx and is known to regulate the E/I balance (Spiegel et al., 2014). At the molecular level, Funahashi et al. (2019) demonstrated that ERK2 phosphorylates NPAS4, which induces the expression of the neurotrophic factor BDNF. Rescue experiments using D1R-MSNs from mice deficient in NPAS4 revealed that MAPK-mediated NPAS4 phosphorylation is required for cocaine-induced reward learning. Additionally, MKL2 was also phosphorylated by cocaine-induced activation of the MAPK signaling pathway (Ariza et al., 2021). Dopamine signaling regulated the interaction between MKL2 and CBP in a phosphorylation-dependent manner. Activation of PKA–MAPK signaling induced the phosphorylation of MKL2 in the nucleus and increased the gene expression of cFOS and NPAS4. MKL2 is known to be a cofactor of the serum response element binding transcription factor (SRF). Although there is no direct evidence linking MKL2 to the E/I balance, depletion of SRF in mature neurons affected epileptogenesis in a mouse model of temporal lobe epilepsy (Losing et al., 2017), suggesting that the MKL2 gene controls the abnormal E/I balance in epilepsy. Thus, the MAPK/ERK pathway can transcriptionally regulate NPAS4 and MKL2 to fine-tune the E/I balance. Consequently, the PKA–RAP1–MAPK signaling pathway, which acts downstream of D1Rs, plays important roles in emotional behavior and learning.

In contrast to D1Rs, D2Rs lead to the inhibition of PKA and DARPP-32 (Nishi et al., 1999). D2Rs work on D2R-MSNs, and their receptors modulate G-protein-coupled inward rectifier potassium (GIRK) channels, which regulate neuronal electrical activity (Mark and Herlitze, 2000). These effects are mediated by GPCRs coupled to Gαi/o proteins. *In vivo* studies have shown the involvement of a β-arrestin2–Akt complex in the D2R signaling pathway in a manner independent of cAMP and G-proteins (Beaulieu et al., 2004; Pack et al., 2018). Akt is activated by its phosphorylation at the threonine 308 (Thr308) residue. The administration of cocaine and amphetamine increased the level of Thr308-phosphorylated Akt in the dorsal striatum (Brami-Cherrier et al., 2002). However, a genetic study in which β-arrestin2 was knocked out revealed that D2Rs inhibit Akt activation through the formation of a signaling complex involving Akt, PP2A, and β-arrestin2. This complex leads to the dephosphorylation of Akt at Thr308 by PP2A, resulting in Akt inactivation (Beaulieu et al., 2005). Further detailed studies of dopamine signaling at different time points after stimulation will be necessary to explain discrepancies in Akt phosphorylation. Despite these discrepancies, there is evidence suggesting that D2R agonists have an anticonvulsant effect, while D2R antagonists increase the risk of seizures (Brodovskaya and Kapur, 2023). In addition, Dunleavy et al. (2013) showed that mice lacking D2Rs exhibit increased susceptibility to seizures. Comprehensive studies of D2R signaling, such as integrated phosphoproteomic and transcriptomic analyses, could provide positive clues to understanding the mechanism underlying neuronal excitability regulation by D2Rs.

Although there are few studies on the molecular mechanism of neuromodulator receptors at presynapses, D2R is known to play a specific role at pre- and postsynapses (Solinas et al., 2019). In mammals, the D2R exists in two isoforms produced by alternative splicing of the *Drd2* gene: the long (D2L) and short (D2S) variants (De Mei et al., 2009). These isoforms differ in their third intracellular loop, with the D2L variant having an insertion of 29 amino acids. D2S, which localizes presynaptically, inhibits dopamine release through the PKA pathway (Ford, 2014). PKA negatively regulates synapsin, a secretory regulatory protein, through its phosphorylation (Menegon et al., 2006). Synapsin II knockout mice show deficits in prepulse inhibition (PPI), impaired habituation to acoustic startle stimuli, decreased social behavior, and increased locomotor activity (Dyck et al., 2009). Taken together with the findings of previous reports, dysregulation of the presynaptic PKA pathway may be involved in abnormal emotional behavior.

## 5 Interplay of dopamine and adenosine signaling for emotional behavior

**Figure 4:**
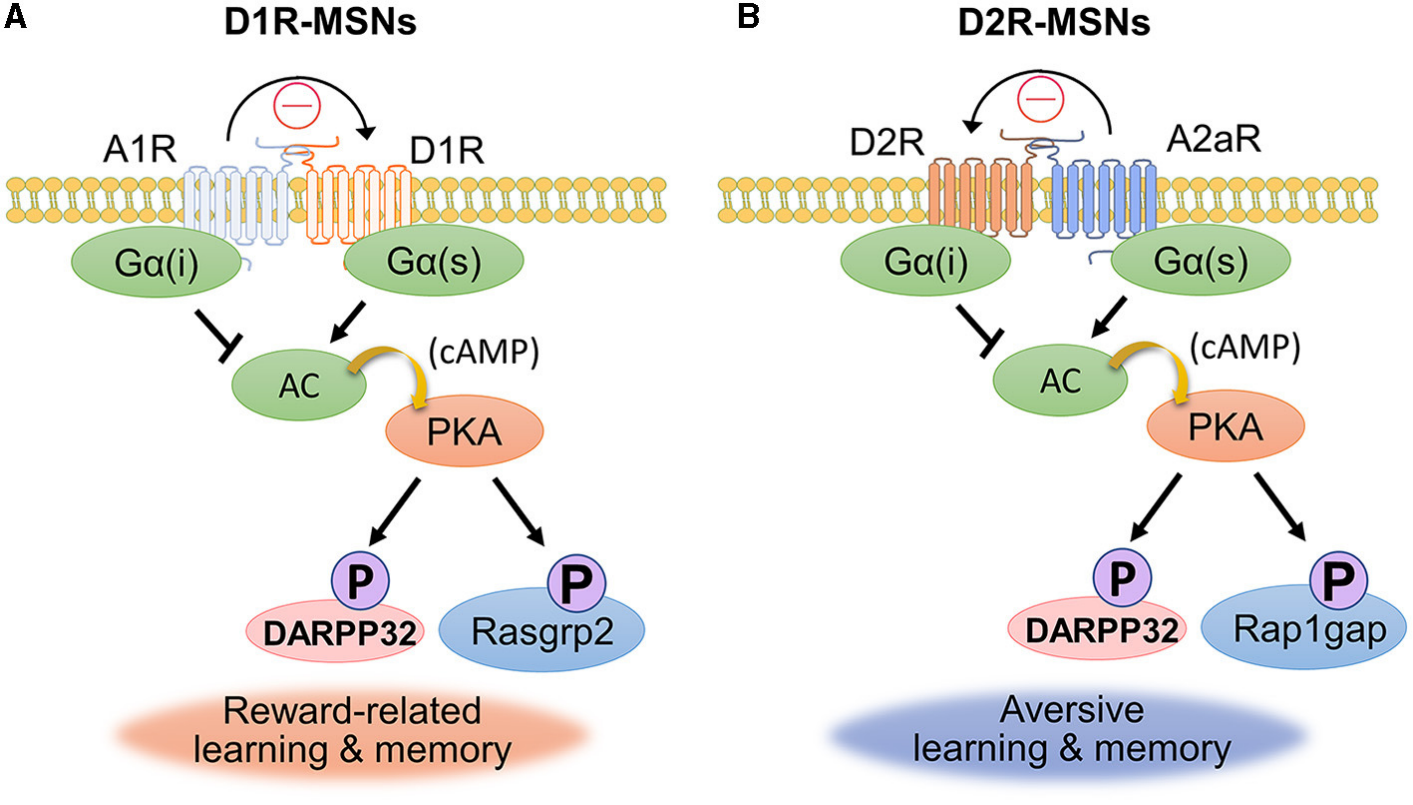
Dopamine and adenosine antagonize the process of cell signaling. **(A, B)** Dopamine and adenosine antagonize each other in diverse physiological responses, and the antagonistic effects are established by both molecular structural actions (curved arrows) and signaling crosstalk (arrows and closed bars). A1Rs are coexpressed with D1Rs in striatonigral neurons **(A)**. A1R/D1R antagonism is involved in reward-related learning and memory. A2aRs are coexpressed with D2Rs in striatopallidal neurons **(B)**. A2aR/D2R antagonism is involved in aversive learning and memory.

Dopamine signaling is strongly influenced not only by the release of its own ligand and by the subtypes of its receptors, but also by the signaling of other neuromodulators. The antagonistic relationships between neuromodulators are crucial for maintaining the balance that allows for optimal brain function. Adenosine, which is known as a neuromodulator, is produced by the breakdown of adenosine triphosphate (ATP) and signals primarily through the A1 (A1R), A2a (A2aR), A2b (A2bR) and A3 (A3R) receptors (Fredholm et al., 2001). A1Rs and A2aRs are highly expressed in the brain, including the cortex, dorsal striatum and hippocampus (Sheth et al., 2014). A2bR and A3R are expressed at lower levels in the brain than are the other two receptors. Adenosine cooperates with dopamine and plays an important role in the regulation of dopamine signaling. Dopamine function in the dorsal striatum and NAc is important for both motor control and the reward system, and adenosine fine-tunes this process (Ferre, 1997). The non-selective adenosine receptor antagonist caffeine is the most widely used psychoactive substance in the world. Studies have shown that caffeine increases extracellular levels of dopamine by antagonizing adenosine A1 receptors (Okada et al., 1997; Solinas et al., 2002). Thus, caffeine increases dopamine concentrations in the brain by blocking adenosine receptors, resulting in arousal, alertness, and motivation. Adenosine also antagonizes dopamine via intracellular signaling pathways. A1Rs are coexpressed with D1Rs in MSNs (Hobson et al., 2013). Alternatively, A2aRs can be coexpressed with D2Rs in MSNs (Ferre et al., 1997). A1R and A2aRs are coupled to Gi and Gs proteins, respectively. As shown in Figure 4, the distribution of receptor subtypes for dopamine and adenosine indicates that dopamine and adenosine have opposing effects on adenylate cyclase activity. Ferre et al. (1998) reported that activation of A1Rs modulates the binding sites of D1R antagonists in cells cotransfected with D1Rs and A1Rs. Indeed, blockade of A1Rs promotes motor activity induced by D1R stimulation (Popoli et al., 1996; Ferre et al., 1998). These pharmacological findings suggest that A1Rs affect D1R availability and activity in this brain region and that antagonism between A1Rs and D1Rs influences the phosphorylation of PKA substrates, such as DARPP-32 and Rasgrp2 (Figure 4A, Kuroiwa et al., 2023).

A2aR/D2R antagonism is also involved in emotional behavior, such as aversion. Aversive behavior refers to actions or responses that are motivated by the desire to avoid or escape from unpleasant or harmful stimuli. It is a complex behavioral response that can be observed in various contexts and is associated with different neuropsychological disorders such as anxiety disorders and posttraumatic stress disorder (PTSD). Aversive behavior is associated with reduced dopaminergic activity in the NAc (McCutcheon et al., 2012), but the molecular machinery underlying aversion remains unclear. A hybrid paper with mathematical and biochemical data explored the role of the PKA–Rap1 pathway in accumbal D2R-MSNs (Zhang et al., 2019). To investigate the interaction between dopamine and adenosine signals, Zhang et al. (2019) developed a mathematical model of antagonism between dopamine and adenosine. This model suggests that the balance between extracellular dopamine and adenosine concentrations determines the phosphorylation level of Rap1gap in D2R-MSNs. Low dopamine levels and above-basal adenosine levels promote Rap1gap phosphorylation, while high dopamine levels and below-basal adenosine levels suppress this process. Based on the results of the *in silico* study, *ex vivo* studies were conducted using pharmacological manipulations of dopamine and adenosine receptors (Figure 4B). The A2aR agonist CGS21680 increased Rap1gap phosphorylation, and pretreatment with the D2R agonist quinpirole blocked this effect in dorsal striatal slices. The D2R antagonist eticlopride increased Rap1gap phosphorylation at the Ser563 residue in D2R-MSNs *in vivo*, and this effect was blocked by pretreatment with the A2aR antagonist SCH58261. These results suggest antagonism between dopamine and adenosine in accumbal D2R-MSNs. Furthermore, in an *in vivo* study, exposing mice to electric foot shocks as an aversive stimulus was combined with monitoring the phosphorylation level of the Ser563-Rap1gap, which activates Rap1 (Lin et al., 2021). The phosphorylation of the Ser563-Rap1gap in accumbal D2R-MSNs was inhibited by pretreatment with A2aR antagonist. An aversive stimulus was found to increase the phosphorylation level of the Ser563-Rap1gap in accumbal D2R-MSNs. Inhibition of PKA, Rap1, or MEK1 in accumbal D2R-MSNs impaired aversive memory in passive avoidance tests, while activation of this pathway potentiated aversive memory. Ahammad et al. (2021) identified a number of proteins, including Rap1gap, whose phosphorylation was promoted by A2aR agonist stimulation. Further phosphorylation signal analysis will help elucidate the regulatory mechanisms of adenosine-induced aversive behavioral learning.

## 6 Interplay of dopamine and acetylcholine signaling for emotional behavior

Acetylcholine enhances excitatory signaling by activating nicotinic acetylcholine receptors localized on the axonal terminals of glutamatergic neurons (Zoli et al., 2018). However, on MSNs and other GABAergic interneurons, acetylcholine can negatively modulate neuronal excitation by activating muscarinic acetylcholine receptors, thereby affecting the E/I balance in the neural circuit (Pancani et al., 2014).

**Figure 5:**
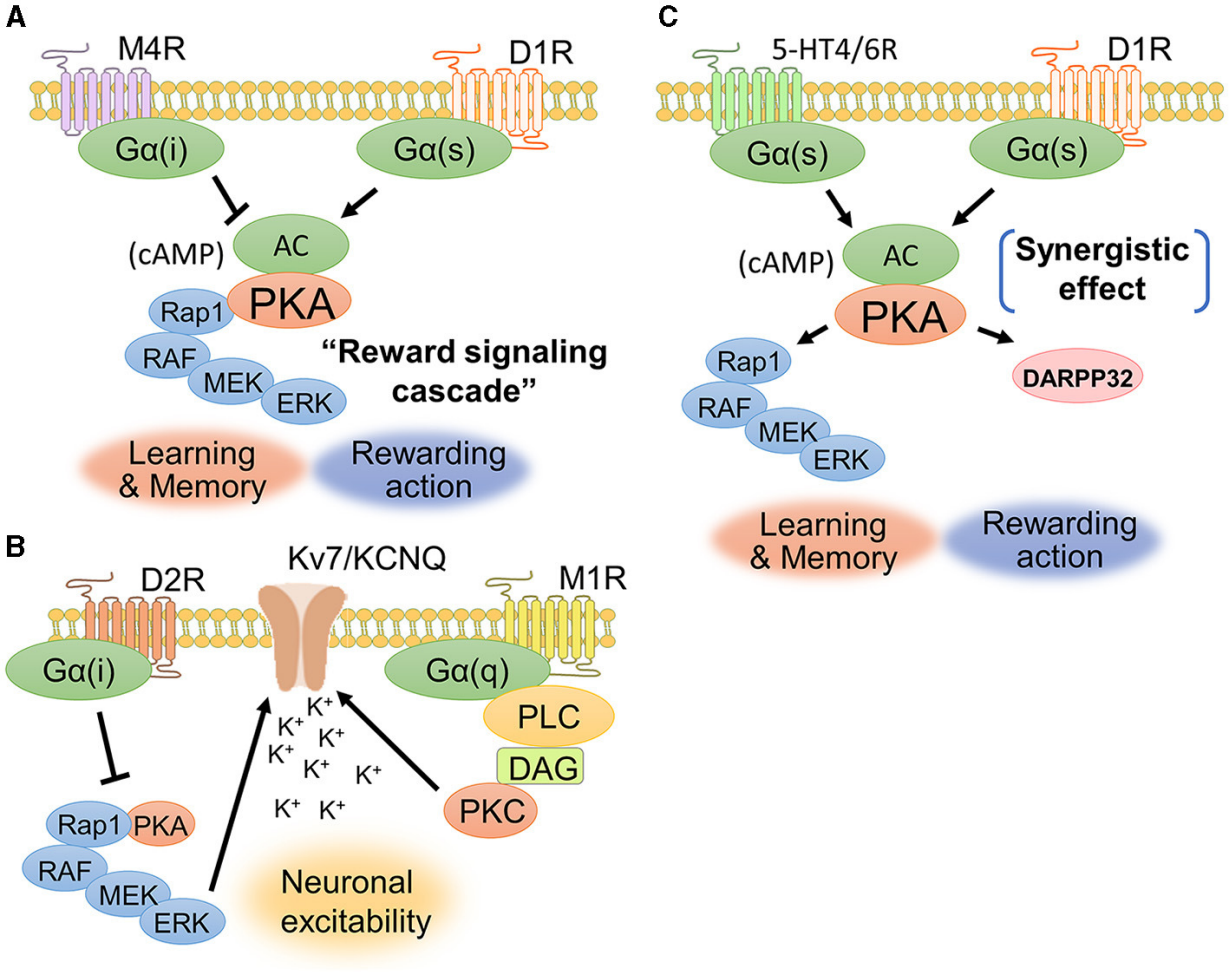
Signaling crosstalks among dopamine, muscarine, and serotonin. **(A)** D1Rs and M4Rs are coexpressed in striatonigral neurons. Activation of D1Rs increases the concentration of cAMP and the phosphorylation level of ERK1/2, while activation of M4Rs is likely to cause a decrease in cAMP production. The cAMP-evoked PKA and its downstream signaling molecules constitute the “reward signaling cascade” because PKA–Rap1–ERK signaling comprehensively regulates cellular responses including membrane excitability, synaptic plasticity, and gene regulation, thereby modulating reward behavior. **(B)** D2Rs and M1Rs are coexpressed in striatopallidal neurons. Acetylcholine enhances the closure of KCNQ2 via Gq-coupled signaling. D2Rs suppress the activity of the PKA–Rap1–ERK signaling pathway. **(C)** D1Rs cooperate with serotonin receptor subtypes, such as 5-HT4Rs and 5-HT6Rs, to activate intracellular signaling. A phosphorylation study of DARPP-32 revealed that the synergetic effect between D1Rs and 5HT4/6Rs contributes to the phosphorylation of DARPP-32.

The interaction between dopamine and acetylcholine is a well-established phenomenon in the brain. Acetylcholine in the dorsal striatum acts antagonistically on dopaminergic transmission and maintains an appropriate balance between acetylcholine and dopamine. The dopamine-acetylcholine balance in nigrostriatal neurons is essential for the regulation of movement and decision-making processes (Chantranupong et al., 2023). For example, D1Rs and muscarinic M4 receptors (M4Rs) are co-expressed in the dorsal striatum (Figure 5A), specifically in striatonigral projection neurons (Guo et al., 2010). M4Rs have been found to modulate D1R-mediated locomotor stimulation (Gomeza et al., 1999). A biochemical study showed that D1R stimulation promotes ERK1/2 phosphorylation, while M4R stimulation is likely to suppress ERK1/2 phosphorylation (Xue et al., 2015). These findings suggest a contradictory relationship between dopamine and acetylcholine in controlling activity of dorsal striatal neuron and behavior.

D2Rs and muscarinic M1 receptors (M1Rs) are coexpressed in striatopallidal projection neurons (Crans et al., 2020). Yamahashi et al. (2022) identified lots of protein kinase C substrates, including KCNQ2, whose phosphorylation was stimulated by acetylcholine. They reported that the application of an electric foot shock or donepezil (an acetylcholine esterase inhibitor), which causes an increase in acetylcholine concentration, induces the phosphorylation of KCNQ2 at Thr217 (Faruk et al., 2022). Phosphorylation of KCNQ2 at Thr217 facilitated the closure of KCNQ channels, thereby increasing neuronal excitability (Surti et al., 2005). Pretreatment with the M1R antagonist VU0255035 suppressed the donepezil-induced phosphorylation of Thr217 in KCNQ2 (Faruk et al., 2022). Following electric foot shock, the cell number and immunofluorescence intensities of Thr217-phosphorylated KCNQ2-positive cells were increased in the NAc of mice. Taken together, these results indicate that acetylcholine released by aversive stimuli enhances the phosphorylation of KCNQ2 at Thr217 via the activation of M1R signaling (Figure 5B). As mentioned in the above section, D2Rs suppress the PKA–Rap1–ERK signaling pathway (namely, the reward signaling cascade) to promote aversive behavior by antagonizing adenosine signaling (Lin et al., 2021). The inhibition of ERK signaling by D2Rs may prevent the phosphorylation of KCNQ channels, resulting in decreased excitability in striatopallidal neurons. KCNQ2 phosphorylation may be a novel drug target for schizophrenia and Parkinson's disease associated with D2R-MSN dysfunction, as it is regulated downstream of dopamine and acetylcholine antagonist pathways.

## 7 Interplay of dopamine and serotonin signaling for emotional behavior

Serotonin activates diverse signaling mechanisms in the dorsal striatum through both G-protein-dependent and G-protein-independent pathways, regulating neuronal excitability and neurotransmitter release. The serotonin receptors are categorized into seven families (5-HT1R to 5-HT7R), each with distinct subtypes and locations (Baez et al., 1995). Both serotonin and dopamine are released in the dorsal striatum and play important roles in reward processing and motivated behavior (Nakamura, 2013). Serotonin and dopamine can have opposing effects on reward behavior, with serotonin generally having inhibitory effects on reward-seeking behavior while dopamine promotes it (Cools et al., 2011). 5-HT1_B_Rs signal through Gi/o proteins to inhibit adenylyl cyclase, decrease cAMP production, and reduce neurotransmitter release in the dorsal striatum (Zhang et al., 2008; Pommer et al., 2021). Additionally, 5-HT2_C_Rs inhibit dopamine release in the dorsal striatum (De Deurwaerdere et al., 2004). 5-HT3Rs are cation ion channels composed of five subunits and are expressed throughout the rodent central and peripheral nervous systems, including in the brainstem, cortex, hippocampus, amygdala, spinal cord, and enteric nervous system (Kilpatrick et al., 1987; Doucet et al., 2000). However, serotonin and dopamine can also act synergistically to promote dopamine release in the dorsal striatum (Figure 5C). Simultaneous serotonin and dopamine release in the dorsal striatum appears to convey highly rewarding stimuli and induces euphoria (Nakamura, 2013). Serotonin can stimulate dopamine release through the activation of serotonin receptors such as 5-HT4Rs and 5-HT6Rs on dopaminergic axons in the dorsal striatum (Steward et al., 1996). 5-HT4Rs are highly expressed in limbic regions such as the hippocampus, amygdala, and prefrontal cortex (Yohn et al., 2017). These findings suggest a synergistic interaction at the level of neurotransmitter release. 5-HT4R agonists can rapidly increase the firing of serotonin neurons (Lucas et al., 2005), suggesting that 5-HT4R activation could be a novel fast-acting antidepressant strategy. In summary, serotonin and dopamine have a complex, bidirectional relationship in the dorsal striatum that underlies their joint role in mediating reward behavior. While sometimes antagonistic, their synergistic actions can augment highly rewarding information and motivate behavior.

## 8 Data-driven study of cell signaling with protein information databases

Protein information databases are useful for exploring and analyzing protein interactions, functional annotations, and biological pathways. These resources are widely used by researchers and scientists to gain insight into the functions of proteins, their interactions, and involvement in various biological processes. BIOGRID and STRING are well-known databases for the study of protein “interactomes.” Each has unique features and functionalities as described below (Box 1).

Box 1 **Interactome-based databases.**

BIOGRID (Biological general repository for interaction datasets):

BIOGRID is a pioneering open-access database that centralizes protein-protein interaction data from biomedical literature (Stark et al., 2006; Oughtred et al., 2021). The advantage of this database is that it is a comprehensive collection of curated and experimentally validated interaction data for molecular interactions in a variety of organisms. This database contains information on physical interactions, genetic interactions, and posttranslational modifications. It provides search capabilities, visualization tools, and data download options to facilitate the exploration and analysis of protein interaction networks.

STRING (Search tool for the retrieval of interacting genes/proteins):STRING is also a widely used protein interaction database that goes beyond just recording protein-protein interactions. It offers a broader perspective by integrating known and predicted associations, including direct (physical) and indirect (functional) interactions (Snel et al., 2000; Szklarczyk et al., 2021). STRING allows users to create customizable protein-protein networks and conduct functional characterization of their gene/measurement sets. Additionally, STRING offers features such as evidence viewers, homology viewer, network clustering methods, and gene set enrichment analysis.

It would be challenging to navigate the complex landscape of signaling because of the limitations of current protein-protein interaction databases. Interactome-based databases are important resources, but they are often inadequate for investigating signal transduction. Protein kinases phosphorylate substrates using ATP, which is abundant in the cell. After transferring the phosphate group to the substrate, the protein kinase rapidly decreases its binding affinity for the phosphorylated substrate and dissociates from the substrate. As the affinity of the protein kinase for the substrate is low compared to the affinity of interactions in protein complex formation (Urh et al., 2009), many kinase–substrate interactions may not be detected in interactome data. The lack of data for kinase-substrate relationships in interactome databases underscores the urgent need for specialized protein information databases that can accurately predict cell signaling. The interactome-based databases, such as STRING and BIOGRID, compile data on the physical and functional relationships between proteins. These databases are particularly useful for understanding the complex network of protein interactions that express cellular responses (Schmidt et al., 2014). Specialized databases, such as KEGG and REACTOME, focus on cataloging and representing molecules and their interactions in biological pathways and processes (Box 2). They provide comprehensive information about reactions, pathways, and biological processes, which is helpful for the analysis of signal transduction, as they provide insights into the specific sequences of biochemical reactions.

Box 2 **Pathway-based databases.**

KEGG (Kyoto encyclopedia of genes and genomes):

KEGG is a comprehensive resource that integrates biological pathway information. It includes a collection of pathway maps, molecular interaction networks, and associated functional annotations (Kanehisa and Goto, 2000; Kanehisa et al., 2012, 2023). KEGG pathways cover various biological processes, including metabolism, cellular signaling, and diseases. The database provides detailed information on genes, proteins, small molecules, and their interactions within specific pathways. KEGG also offers analysis tools and data visualization options to aid in the interpretation of pathway data.

REACTOME:

REACTOME is a free, open-source, and peer-reviewed pathway database that aims to provide a comprehensive overview of many human biological processes (Joshi-Tope et al., 2005; Milacic et al., 2023). Its goal is to provide intuitive bioinformatics tools for the visualization, interpretation, and analysis of pathway knowledge. The navigation menu of the Reactome website includes the sections “About,” “Content,” “Docs,” “Tools,” and “Community,” with each subsection providing more specific information and resources related to Reactome. In addition, pathway analysis can be easily performed by entering any gene set.

One of the key strengths of the KEGG database is its extensive data coverage. It provides a vast array of biological information, from genomic and molecular data to large-scale molecular data on biological systems, diseases, drugs, and chemical substances. Although KEGG strives to maintain accuracy, it is not immune to errors or inconsistencies due to the vast amount of data it contains. Researchers must cross-verify the information with other resources to ensure its accuracy. The data in REACTOME have been curated and peer-reviewed by expert biologists, ensuring that the information is reliable and accurate. Perhaps the most representative of the strengths of the REACTOME phosphorylation database is its user-friendly interface, allowing users to manipulate data dynamically and facilitate the exploration of complex biological pathways. While REACTOME covers a wide range of biological processes, it may lack the details required for specific phosphorylation events. Some researchers might need more specialized resources. Recently, to support more detailed signal transduction analysis, additional databases (e.g., KANPHOS, Scop3P) that register structured data specific to certain types of information (phenomena) have been created (Ramasamy et al., 2020; Savage and Zhang, 2020; Ahammad et al., 2021).

## 9 Conclusion

This review explores the complex interactions between neuromodulators like dopamine, acetylcholine, and serotonin, and their crucial roles in synaptic transmission and cell signaling. The E/I balance, which is mediated by neurotransmitters such as glutamate and GABA, is essential for a healthy brain. Perturbations in this balance contribute to the pathophysiology of neurodevelopmental and psychiatric disorders, such as ASD and schizophrenia. Despite the pivotal roles of neuromodulators, our understanding of their molecular mechanisms is still developing. The reason is that neuromodulators function in multiple regions of the brain in diverse manners. To better understand how cell signaling pathways in each brain region control motor function, reward, motivation, and learning, comprehensive phosphorylation studies at the whole-brain level are needed. One approach is focused proteomics, which uses phosphorylation motifs and anti-phosphorylation antibodies to search for phosphosubstrates for protein kinases. The KiOSS method, developed by Nishioka et al. (2019) combines phosphatase and kinase inhibitors and uses phosphorylation binding motifs to enrich for phosphopeptides. This method has been successful in identifying novel substrates downstream of dopamine, adenosine and acetylcholine. However, there are still challenges ahead. In experiments focusing on a small number of proteins, understanding the causal relationships between phosphorylation variation and brain function would take an enormous amount of time. A data-driven approach can enable a broad investigation of phosphorylation-mediated signaling network. An approach that not only focuses on the phosphorylation events of individual proteins but also takes a macroscopic view as a phosphorylation network will be necessary to identify new signaling pathways, discover new phosphorylation sites, and attempt to elucidate the complex signaling network. In the field of cancer research, the analysis of phosphorylation signaling data using bioinformatics technologies is a powerful driving force. An example of a data-driven approach is the analysis of phosphoproteomic data using a novel bioinformatics tool, kinase-substrate enrichment analysis (KSEA) (Casado et al., 2013). KSEA allowed researchers to identify leukemia cells, revealing heterogeneity in pathway activation in cells and leading to the development of more effective therapies. This example shows how valuable insights and advances in phosphorylation signaling analysis can be achieved through data-driven research. Researchers can uncover the complex and dynamic nature of phosphorylation signaling and pave the way for the development of new strategies for disease diagnosis, prognosis, and treatment by using high-throughput technologies and bioinformatics tools.

## Author contributions

DT: Conceptualization, Funding acquisition, Validation, Writing – original draft, Writing – review & editing. TN: Conceptualization, Funding acquisition, Validation, Writing – review & editing. JY: Conceptualization, Funding acquisition, Writing – review & editing. KK: Conceptualization, Funding acquisition, Validation, Writing – review & editing.
